# Evaluating electronic cigarette cytotoxicity and inflammatory responses in vitro

**DOI:** 10.18332/tid/147200

**Published:** 2022-05-10

**Authors:** Indu Sinha, Reema Goel, Zachary T. Bitzer, Neil Trushin, Jason Liao, Raghu Sinha

**Affiliations:** 1 Department of Biochemistry and Molecular Biology, Penn State College of Medicine, United States; 2 Department of Public Health Sciences, Penn State College of Medicine, United States

**Keywords:** electronic cigarettes, e-liquids, flavors, inflammatory response, cytotoxicity

## Abstract

**INTRODUCTION:**

Cigarette smoking poses many health risks and can cause chronic obstructive pulmonary disease (COPD), cardiovascular disease, cancer of the lung and other organs. Smokers can substantially reduce their risks of these diseases by quitting, but nicotine addiction makes this difficult. Alternatives, such as electronic cigarettes (e-cigarettes), may provide a similar dose of nicotine, but expose users to fewer toxic chemicals than traditional cigarettes and may still be harmful especially for dual users, therefore, we sought to develop bioassays that can assess the potential toxicity and inflammatory response induced by e-cigarette liquids (e-liquids) with and without flavors.

**METHODS:**

E-liquids with varying nicotine content and flavors were aerosolized through growth media and exposed to human bronchial epithelial cell line (BEAS-2B) and human monocyte-macrophage cell line (THP-1) *in vitro*. Cytotoxicity in response to e-cigarette aerosols was measured by MTT assay in BEAS-2B cells and inflammatory response was measured by TNF-α, IL-6, IL-8, and MCP-1 released from THP-1 cells. In addition, the oxidative stress marker, REDD1, and impact on phagocytosis, was assessed following exposure of BEAS-2B and THP-1 derived macrophages, respectively. Cigarette smoke extract was used as a positive control with known cytotoxicity and impairment of inflammatory response.

**RESULTS:**

E-cigarette aerosols induced moderate cellular toxicity in bronchial epithelial cells. Our data also show that low nicotine levels are less damaging to the bronchial epithelial cells, and flavors in e-liquids influence the combined inflammatory response markers, phagocytosis, and REDD1 when examined *in vitro*.

**CONCLUSIONS:**

Our *in vitro* bioassays can be utilized to effectively measure flavor and nicotine-induced effects of e-cigarettes on combined inflammatory response and cytotoxicity in human macrophages and human bronchial epithelial cells, respectively.

## INTRODUCTION

Chronic obstructive pulmonary disease (COPD) represents an increasing burden of disability as well as leading cause of death worldwide and is projected to be the seventh leading cause of disability and fourth leading cause of death by the year 2030^1^. Smoking is the primary cause of COPD, which is defined as a common preventable and treatable disease demonstrating persistent and progressive airflow limitation and enhanced chronic inflammatory response in the airways and the lung due to noxious particles or gases^2^. In addition, increased oxidative burden plays an important role in the pathogenesis of COPD^3,4^. Smoking cessation in early stages of COPD can be beneficial and has been found to reduce the rapid decline of ventilatory function in smokers. Early diagnosis of COPD in asymptomatic smokers may motivate smokers to attempt smoking cessation, and delay its progression to a more advanced stage^5^, or reduce mortality^6^. However, smoking cessation can be difficult to achieve, especially among those with higher nicotine dependence^7,8^. Since nicotine is highly addictive, smoking tobacco products containing nicotine often becomes a lifelong habit. Although electronic cigarettes (e-cigarettes) are not an FDA approved nicotine replacement therapy (NRT), switching from combustible tobacco products to e-cigarettes may be considered a useful alternative for smokers with COPD, but more evidence is required to establish potential harm reduction. A recent review describes the experimental and clinical evidence of e-cigarette toxicity and its deleterious health effects^9^.

There are several types of e-cigarettes and thousands of varieties of e-liquids available on the market. E-liquids are mainly composed of nicotine, propylene glycol (PG) and/or vegetable glycerin (VG), and various flavors (e.g. tobacco, menthol/mint, fruit, dessert/sweets, alcohol, nuts/spices, candy, coffee/tea). The ability of e-cigarette users to choose from various flavors has been shown to be an important factor to users^10^. While the majority of flavor compounds are considered to be generally recognized as safe (GRAS) by the FDA, this designation is intended for oral ingestion only, and not for inhalation exposures. Thus, more inhalation exposure studies are needed to assess the effects of e-liquid flavor compounds.

Studies to determine the impact of aerosolized e-liquids on the bronchial epithelial cells, inflammatory response markers, and the influence on oxidative stress, may provide evidence for the potential harmful effects of e-cigarettes. Cigarette smoke extract (CSE) was used as a positive control with known cytotoxicity to bronchial epithelial cells and impairment of inflammatory response. REDD1, an oxidative stress response protein, is strongly induced by CSE and is responsible for some of the pathology reported in cigarette smokers with COPD^11^. To examine the possible harmful effects, we used growth media exposed to aerosolized e-liquids with and without flavors or to CSE (positive control), to treat immortalized human bronchial epithelial cells (BEAS-2B), NCI-H460 cells, and differentiated monocytic cells (THP-1). These cell types were chosen to evaluate the impact of e-cigarettes on human lung epithelial cells and macrophages. We selected a few flavors for our study. Menthol being a popular flavor among the youth and adults^12^ and a series of vanilla flavors as well as ‘rainbow candy’ which were earlier evaluated for free radical generation^13^. Following the treatments with these e-liquids, we measured cytotoxicity and oxidative stress marker (REDD-1) along with inflammatory response markers (TNF-α, IL-6, IL-8, and MCP-1) in respective cell types.

## METHODS

### E-liquids

E-liquids were either prepared from pure humectants (PG and VG; Sigma-Aldrich, St. Louis, MO) or were purchased from NiQuid (NiQuid.com, Miamisburg, OH). E-liquids from NiQuid contained 0, 6 or 12 mg/mL nicotine with and without Smoothol flavor. The Smoothol flavor potentially imparts a fresh, strong, and minty taste with a hint of cream and sweetness. Other flavors: vanilla, vanilla custard, rainbow candy and french vanilla were purchased from NicVape.com (Spartanburg, SC). These 4 flavors were selected from a list of 49 distinct flavors that were evaluated for free radical generation in aerosolized e-liquids^13^. Additionally, an e-liquid containing 36 mg/mL nicotine and US menthol flavor (LifeSmoke, York, PA) was included to test the effect of directly adding e-liquid to cell culture medium for one experiment. All the commercial e-liquids were based on 60:40 ratio for PG:VG, unless stated otherwise.

### E-cigarette device and vaping protocol

E-liquids were aerosolized at 3.6 volts using 2.5 Ω bottom coil in an e-cigarette (Innokin iTaste VV4 v4) for 30 puffs (4 s duration each with 60 s inter-puff interval). An in-house designed and built switching device was used for vaping as described previously^14^. Careful consideration was given to avoid dry puffing during a session by preloading the cartomizer 5 min prior to vaping, and allowing 4 puffs to be passed through PBS and ensuring that aerosol generation is visible. Cartomizers were weighed before and after puffing to measure the e-liquid consumption and aerosol production. The aerosols were collected in an impinger containing 20 mL cell growth medium connected through house vacuum via a flow meter. Typically, about 200 mg of e-liquid was vaped using these conditions at 0.5 L/min, resulting in a 1% solution in medium. The different cell types were incubated with the 1% solution in growth medium to measure cytotoxicity, oxidative stress, inflammatory response markers, and phagocytosis.

### Research cigarette smoke extracts

Mainstream cigarette smoke was generated by a single-port smoking machine (Human Puff Profile Cigarette Smoking Machine (CSM-HPP), CH Technologies, NJ, USA) using the 3R4F research cigarettes (University of Kentucky, USA) or very low-nicotine SPECTRUM 102 (National Institute of Drug Abuse). The smoke was collected through an impinger (containing 20 mL growth medium for BEAS-2B or THP-1 cells). For cigarette smoke extract (CSE), 30 puffs were collected using same profile as e-cigarettes (4 s duration each with 60 s inter-puff interval). For treating cells, this solution was considered as 100% CSE and BEAS-2B and THP-1 derived macrophages were incubated for 3–24 h to measure cytotoxicity and inflammatory response markers, respectively.

### Nicotine content recovery from cigarette smoke and e-liquids

Nicotine was measured by HPLC/UV analysis in commercial e-liquids and in growth media through which aerosolized e-liquids had been passed, as well as CSE from 3R4F and SPECTRUM 102. The analysis was accomplished on a Phenomenex Synergi Max RP column (4.6 × 250 mm) using a Shimadzu 10ADvP HPLC system consisting of two pumps, an autosampler and an ultraviolet detector set to 259 nm. Data were recorded on a Hitachi D2000 integrator. Injection volumes were 10 or 20 μL for each sample. HPLC solvent A was 0.01% ammonium hydroxide in water, solvent B was methanol. The elution program was a 1-minute hold at initial conditions of 95% solvent A, followed by a 30-minute gradient to 95% solvent B, held for 5 minutes, before returning to initial conditions. Using this program nicotine eluted at approximately 27 minutes. The flow rate was 1 mL/min and the column was equilibrated at initial conditions for 10 minutes prior to each injection. For quantification, a standard curve of nicotine dissolved in media at different concentrations (range: 0.5–5 μg/mL) was generated.

### Cell lines

Immortalized human bronchial epithelial cells (BEAS-2B) were purchased from ATCC (Manassas, VA) and maintained in Bronchial Epithelial Cell Medium (BEBM) with growth supplements (Lonza, Walkersville, MD). BEAS-2B cells require special coating on the flasks with a mixture of 0.01 mg/mL fibronectin (Sigma, St. Louis, MO), 0.03 mg/mL bovine collagen type I (Advanced Biomatrix, San Diego, CA) and 0.01 mg/mL bovine serum albumin (Gemini Bio-Products, West Sacramento, CA) dissolved in BEBM. NCI-H460 cell line was purchased from ATCC and maintained in RPMI-1640 with 10% Fetal Bovine Serum (Gemini Bio-Products, West Sacramento, CA), 1% streptomycin-penicillin (Invitrogen, Carlsbad, CA). Human monocytic cell line (THP-1) was purchased from ATCC (Manassas, VA) and maintained in RPMI-1640 supplemented with heat inactivated 10% FBS (Gemini Bio-Products, West Sacramento, CA). All the cell types were maintained and treated at 37°C in a humidified incubator in presence of 5% CO_2_. Each cell line was maintained in culture for only 4–5 passage numbers for a given experiment. Short Tandem Repeat (STR) DNA profiling was used to confirm the authenticity of each cell line.

### Cytotoxicity (MTT assay)

Cytotoxicity was measured in BEAS-2B and NCI-H460 cells following treatments for MTT assay as described earlier^15^. Briefly, these cell types (1×10^4^/well) were plated on pre-coated 96-well plates for 24 h and treated with 1% solution of e-cigarette aerosols in growth medium (with or without nicotine/flavor or with CSE using several concentrations (100%, 50%, 40%, 30%, 20%, 15%, 10%, 5%, 1%, 0.5%, 0.1%) for another 24 h followed by MTT assay. For MTT assay cells were treated with 3-(4,5-dimethylthiazol-2-yl)-2,5-diphenyltetrazolium bromide (MTT, Sigma, St. Louis, MO) (50 μg/well) for 3 h in dark at 37^ο^C. MTT solution was removed from the wells and was replaced by 100 μL of DMSO/well to dissolve the purple blue formazan particles and the plate was read at 570 nm with correction at 630 nm in Molecular Devices plate reader. MTT assay was performed in triplicate for each treatment.

### Evaluating inflammatory response markers

THP-1 cells were differentiated following modification of the protocol described previously^16^. Briefly, 2.5 × 10^5^ cells/well were plated in 24-well plates in the presence of 100 nM Phorbol 12-myristate 13-acetate (PMA; Sigma, St. Louis, MO) for 3 days, followed by a rinse with PBS and incubation with 1% e-cigarette aerosol infused media (with and without nicotine/flavors) or CSE in presence of LPS (100 ng/mL, Sigma, St. Louis, MO) for 6 h. Spent media were frozen at -20°C until used for measuring inflammatory response markers by ELISA. The spent media were diluted 1:1 in assay diluent and TNF-α, IL-6, IL-8 and MCP-1 amounts were determined using Quantikine^TM^ ELISA Kits (R&D Systems, Minneapolis, MN) following the manufacturer’s instructions.

### Phagocytosis assay

THP-1 cells were differentiated into macrophages by 100 nM PMA for 2 days at 37°C and incubated with media infused with 1% e-cigarette aerosol solution (PG:VG, 60:40) with and without nicotine and Smoothol (NicQuid) in presence of fluorescein-labeled rabbit IgG-coated latex beads (Cayman Chemical, Ann Arbor, MI) for an additional 3 h following manufacturer’s instructions. Macrophages were then treated with 0.04% solution of trypan blue for 2 min to remove the non-internalized latex beads and cells were finally rinsed with PBS prior to observation under fluorescent microscope (Olympus, Center Valley, PA). A total of 8–10 high-power fields (400X) were used to count macrophages and the number of macrophages showing internalized fluorescent latex beads divided by total macrophages (about 500) was estimated as the phagocytic index.

### Quantification of REDD1 protein levels

BEAS-2B cells were incubated with growth medium (control) or 1% aerosolized e-liquids (with and without flavor) or growth media with 100% CSE of 3R4F (Research Cigarette) at 37°C for 4 h. A separate flask of BEAS-2B cells were incubated in growth medium and exposed to 1% oxygen environment in a sealed chamber at 37°C for same amount of time. The cells were collected by scraper, washed in cold PBS and lysed in RIPA buffer (Sigma, St. Louis, MO) containing protease inhibitors. Equal amounts of protein (50 μg) were run for all the samples in SDS-PAGE and western blot was performed on the proteins transferred onto nitrocellulose membrane as described earlier^17^. Primary antibody against REDD1 (Proteintech, Chicago, IL) and β-actin (Santa Cruz Biotechnology, Santa Cruz, CA) were reacted separately at 1:1000 and 1:4000 dilution, respectively, with the blot. The HRP-conjugated anti-rabbit and anti-goat secondary antibodies (Cell Signaling, Danvers, MA) were incubated at a dilution of 1:3000. Band expressions were developed using Pierce^TM^ ECL reagents (Thermo Scientific, Rockford, IL) and band densities were quantified by Image J analysis (National Institute of Health, Bethesda, MD). Fold change in band densities of REDD1 protein were normalized to band density of β-actin for all the samples.

### Statistical analysis

All the assays in the study were conducted at least twice. Analyses were performed using the statistical software SPSS (version 13.0, SPSS) and data were expressed as means ± SD. Differences between means were evaluated using 1-way ANOVA and differences among treatment means were assessed using Tukey’s test. Differences were considered significant at p<0.05. Effects of flavors in aerosols were compared with PG:VG for cytotoxicity and with PG:VG and LPS alone for inflammatory cytokines using Wilcoxon rank sum tests. For phagocytosis index, Wilcoxon rank sum test was applied to compare media alone and PG:VG with PG:VG (nicotine), PG:VG (nicotine + Smoothol) and PG:VG (Smoothol) containing aerosols reacted with THP-1 derived macrophages. To determine an impact of flavors together on combined inflammatory response cytokines (TNF-α, IL-6, IL-8 and MCP-1), the data sets were converted to Z-score within each biomarker to achieve uniform scales. Comparisons of means for combined inflammatory response cytokines between LPS treatment alone and PG:VG aerosols + LPS were made with flavored aerosols in presence of LPS by ANOVA followed by Tukey’s multiple comparison test. Differences were considered significant at p<0.05. The IC_50_ was estimated using a 4-parameter logistic dose-response curve as implemented in R package *drc*.

## RESULTS

**Figure 1:**
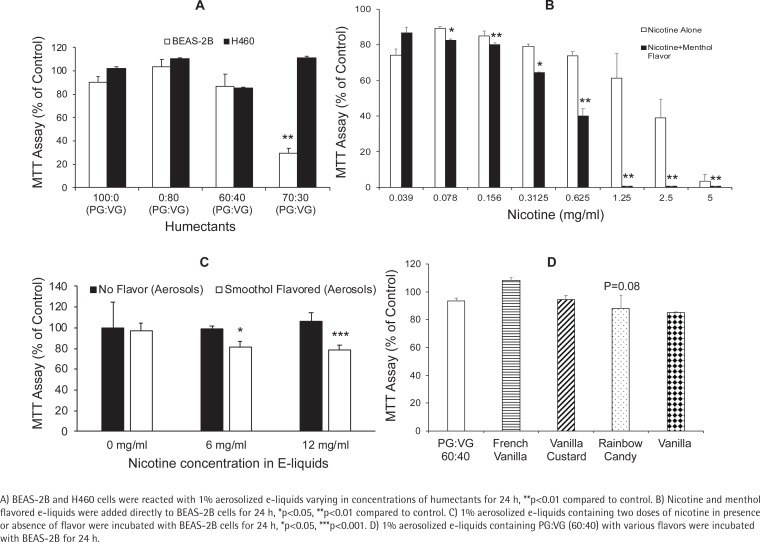
Impact of humectants, nicotine and flavors on cytotoxicity measured by MTT assay

Aerosolized humectants influenced the proliferation of bronchial epithelial (BEAS-2B) cells and lung cancer cells (H460). Cytotoxicity, as measured by MTT assay, was observed for PG:VG at 70:30 ratio (p<0.01) in BEAS-2B cells, but there was no effect on H460 cell growth for the same PG:VG ratio (Figure 1 A). Since PG:VG ratio of 60:40 had moderate cytotoxic impact on both lung cell types and is one of the common humectant choices among e-cigarette users, and our focus was to evaluate impact of e-liquids in a normal setting, we selected this ratio for the remaining experiments in BEAS-2B cells only for our study.

### Impact of nicotine and flavors on BEAS-2B cell growth

In order to observe the impact of nicotine and flavors on growth, we directly added nicotine to e-liquid (PG:VG, 60:40) or added a nicotine-containing menthol flavored e-liquid (PG:VG, 60:40) directly to growth medium of BEAS-2B cells. A dose-dependent decrease in cell growth was observed by both nicotine and nicotine + menthol flavored e-liquids (Figure 1 B). The IC_50_ dose for nicotine alone was 2.04 mg/mL and for nicotine + menthol flavor was 0.53 mg/mL, thus indicating the pronounced cytotoxic effect of the flavor directly added to BEAS-2B cells in culture.

When 1% aerosolized e-liquids in growth medium were incubated with BEAS-2B cells for 24 h, the cell growth was significantly reduced for Smoothol-containing nicotine e-liquids in a dose-dependent manner (Figure 1 C). However, aerosolized e-liquid containing nicotine alone did not have an appreciable impact on BEAS-2B cells. Based on the recovery of nicotine from the original e-liquids and the aerosolized e-liquids into growth medium, with and without Smoothol, we were able to recover 66–81% of nicotine (data not shown) in the growth medium or original e-liquids on mg/mL basis. Therefore, the nicotine concentrations presented in the figures are representing approximately 0.66–0.81% of the added amounts to the cell types.

Further analysis of 1% aerosolized e-liquids with additional flavors (without nicotine) in PG:VG (60:40) differed in their cytotoxic effects on BEAS-2B cells (Figure 1 D) compared to control (no treatment). Specifically, ‘rainbow candy’ and vanilla showed a moderate decrease in BEAS-2B cell growth compared to PG:VG alone.

We also determined the impact of CSEs from 3R4F and Spectrum 102 cigarettes in BEAS-2B cells (Supplementary file Figure 1). As expected, dose-dependent cytotoxicity in BEAS-2B cells was observed for varying concentrations of CSE in growth medium and was greater for 3R4F compared to Spectrum 102. When comparing cell growth of BEAS-2B incubated with 1% aerosolized e-liquids versus CSE, it was clear that CSE is much more cytotoxic based on exposure equivalent of 30 puffs each (Figure 1 C, and Supplementary file Figure 1) even after considering growth inhibition due to Smoothol flavor.

### Nicotine and flavor influence inflammatory response markers

We determined TNF-α, IL-8, IL-6 and MCP-1 levels following treatments with aerosolized e-liquids of varying nicotine concentrations with and without Smoothol in THP-1 derived macrophages for 6 h in presence of LPS. This particular incubation time was selected based on earlier determination of optimum LPS response for TNF-α in time-lapsed study ranging from 1h to 24 h incubations for PMA-induced differentiation of THP-1 cells (Supplementary file Figure 2).

TNF-α levels in THP-1 derived macrophages were significantly reduced by Smoothol flavored aerosolized e-liquid (p<0.05) compared to unflavored aerosolized e-liquid in the absence of nicotine (Supplementary file Figure 3 A). Moreover, Smoothol flavored aerosolized e-liquid showed a moderate decrease (p>0.05) in IL-8 levels when compared to unflavored aerosolized e-liquid in the absence of nicotine (Supplementary file Figure 3 B). Further, the IL-6 levels were significantly reduced by aerosolized Smoothol (p<0.05) compared to unflavored e-liquid in absence of nicotine (p<0.05) (Supplementary file Figure 3 C). Nicotine in 1% aerosolized unflavored e-liquids reduced IL-6 levels at 6 mg/mL dose compared to LPS (p<0.05), while 1% aerosolized Smoothol in presence of 6 mg/mL nicotine increased IL-6 level (p=0.05) compared to unflavored e-liquid containing 6 mg/mL nicotine. The MCP-1 levels in THP-1 derived macrophages were reduced (p>0.05) by Smoothol flavored e-liquid (no nicotine) compared to unflavored aerosolized e-liquid. In addition, levels of MCP-1 showed greater decrease with nicotine in unflavored aerosolized e-liquids (p>0.05) (Supplementary file Figure 3 D).

**Figure 2:**
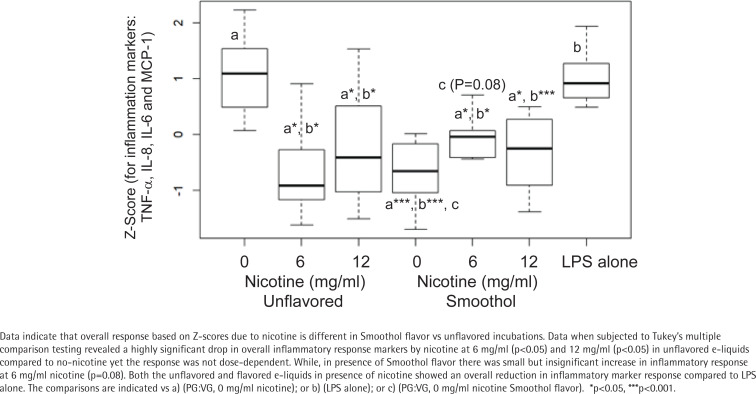
Combined inflammatory response biomarkers in THP-1 derived macrophages following incubation with or without 1% aerosolized nicotine-containing e-liquids

Since we were interested in evaluating the combined inflammatory response of e-liquids towards the cytokines/chemokine and their determined levels varied, a Z-score was computed for all four inflammatory response markers (TNF-α, IL-8, IL-6, and MCP-1) combined. A significant reduction was observed in combined inflammatory response by 1% aerosolized Smoothol-containing e-liquid compared to aerosolized PG:VG (60:40) (Figure 2). Furthermore, comparing nicotine vs PG:VG (60:40) there was a significant reduction in combined inflammatory response by 6 mg/mL and 12 mg/mL concentrations. In addition, both the unflavored and Smoothol flavored nicotine-containing aerosolized e-liquids showed an overall reduction in inflammatory marker response compared to LPS alone. Since the flavored vs unflavored nicotine-containing aerosols differed in their combined inflammatory response markers, we performed an interaction analysis which revealed a greater significance for only unflavored e-liquids, and no effect was observed for Smoothol flavor in the nicotine-containing aerosolized e-liquids. Thus, indicating a potential role for nicotine in unflavored e-liquid for suppressing inflammatory response markers.

To further delineate impact of flavors on inflammatory response markers, several flavors including vanilla, french vanilla, ‘vanilla custard’ and ‘rainbow candy’ were reacted in their 1% aerosolized e-liquids forms with the THP-1 derived macrophages under same conditions as the above experiment. Results on TNF-α, IL-8, IL-6, and MCP-1 indicate that ‘rainbow candy’ showed a trend towards reducing TNF-α levels in THP-1 macrophages (Supplementary file Figure 4 A). None of the flavors significantly impacted the IL-8 (Supplementary file Figure 4 B) and IL-6 levels (Supplementary file Figure 4 C). However, aerosolized e-liquids with all the flavors, except vanilla, showed a significant (p<0.05) reduction in MCP-1 levels when compared to LPS alone (Supplementary file Figure 4 D). The flavor ‘rainbow candy’ had a similar effect on all the inflammation markers.

**Figure 3:**
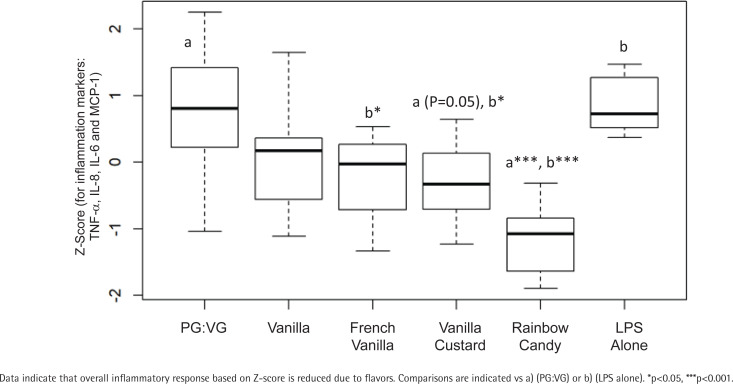
Combined inflammatory response biomarkers in THP-1 derived macrophages following incubation with 1% aerosolized flavored e-liquids

Upon compiling the Z-score for all 4 inflammatory response markers combined for different flavors, there was a significant reduction for treatments with ‘vanilla custard’ and ‘rainbow candy’ compared to PG:VG alone. Furthermore, a reduction after treatment with these flavors was also observed compared to LPS alone (Figure 3).

Additionally, CSEs from very low-nicotine Spectrum 102 as well as 3R4F cigarettes were reacted with THP-1 derived macrophages to estimate TNF-α levels in presence of LPS for 6 h. Comparing serial concentrations of CSEs for inflammatory response, 3R4F and Spectrum cigarettes showed a dose-dependent increasing trend in TNF-α release from THP-1 derived macrophages (Supplementary file Figure 5) until the 15% and 30% dilution, respectively (only moderate cytotoxicity was observed at this dilution in BEAS-2B cells). Beyond the 15% and 30% CSE dilutions in 3R4F and Spectrum cigarettes, there was a decline in TNF-α levels. The strong reduction in TNF-α by CSE at higher concentrations could also be due to cell death that was not determined in this experiment.

### Impact of aerosolized e-liquids on oxidative stress in BEAS-2B cells

**Figure 4:**
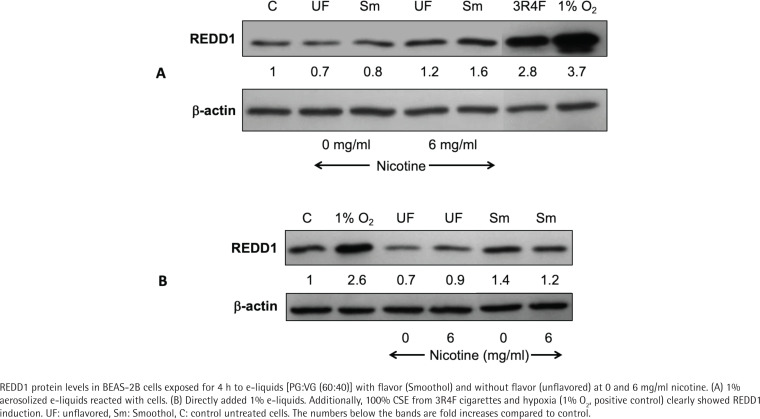
E-liquids impact REDD1 in BEAS-2B cells

We examined the effect of 1% aerosolized e-liquids on REDD1 protein levels in BEAS-2B cells and observed an elevated REDD1 (1.6-fold) in Smoothol flavor at 6 mg/mL nicotine (Figure 4 A). In contrast, 3R4F CSE (100%) showed a higher increase (2.8-fold) in REDD1 levels. In order to determine whether Smoothol flavor is truly influencing REDD1, we repeated the experiment by exposing the BEAS-2B cells directly to 1% e-liquids, and the results showed that Smoothol flavor did increase REDD1 protein levels by 1.2 in no-nicotine and 1.4-fold in 6 mg/mL nicotine e-liquids (Figure 4 B).

### E-liquids influence phagocytic activity of THP-1 derived macrophages

**Figure 5:**
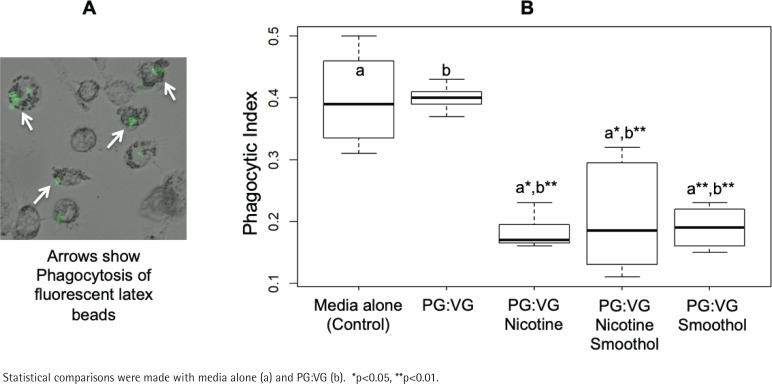
Aerosolized e-liquids influence phagocytic activity of macrophages. (A) Representative THP-1 derived macrophages showing engulfed fluorescein-labeled rabbit IgG-coated latex beads. (B) Phagocytic index for THP-1 derived macrophages treated with 1% e-liquids (PG:VG, 60:40) with or without nicotine (6 mg/mL) and with or without flavor (Smoothol)

Aerosolized nicotine-containing 1% e-liquid with and without Smoothol flavor were reacted with THP-1 derived macrophages in the presence of fluorescent beads for 3 h in order to measure phagocytic activity (Figure 5 A). Results suggested that Smoothol flavor alone, nicotine alone, as well as combined Smoothol and nicotine, reduced the phagocytic index by 52.5%, 55% and 47.5%, respectively, compared to media alone or PG:VG alone in THP-1 derived macrophages (Figure 5 B).

## DISCUSSION

The majority of the COPD smokers suffering from emphysema and/or chronic bronchitis are willing to switch to e-cigarettes and generally improved symptoms have been reported, even displaying pulmonary harm reversal^18^. In a 5-year follow-up study of COPD, e-cigarette users had a significant diminution in COPD exacerbations and significant constant improvements in lung function, CAT scores, and 6-minute walk distance, compared with the reference group^19^. Despite this, the literature varies; a study showed a reduction of forced expiratory volume in 1 s (FEV1) in never smokers when compared with FEV1 following acute and passive e-cigarette exposures^20^ and low toxicity of e-cigarettes *in vitro*^21^, while others find higher toxicity and inflammatory effects of e-cigarettes *in vitro*^22-27^ and *in vivo*^24,28,29^.

There is a need to develop an *in vitro* bioassay system to help inform FDA about the relative toxicity of e-cigarettes in general public and more so for COPD smokers. We therefore sought to perform a systematic investigation of e-cigarette cytotoxicity, oxidative stress and inflammatory responses using BEAS-2B and THP-1 cell *in vitro*.

With hundreds of e-liquids currently available containing variable ratios of humectants, nicotine and flavor combinations, determining the relative toxicity of e-cigarette aerosols has proven especially challenging. E-cigarette flavors remain popular and are incorporated into a multitude of products, despite regulatory restrictions. Even though the FDA is interested in research that shows benefits of reducing nicotine content in tobacco products, there are no clear guidelines on the relative amounts of other components in e-liquids, including flavors. More recently, an online survey study found that flavor-associated adverse reactions, such as respiratory irritations, were reported by 6.9% participants^30^.

Our data indicate that PG:VG at 60:40 was not toxic to BEAS-2B while 70:30 ratio was growth inhibitory (Figure 1 A). Similar findings were reported elsewhere^25^. Even though we did not find any effect of PG:VG on H460 cells, others have reported growth inhibition on various lung cancer cell lines^25,26,31^. We believe that for investigating impact of e-cigarettes in lungs, it is better to experiment on near normal bronchial epithelial cells and so we performed other assays using BEAS-2B cells. Menthol flavor with added nicotine was more toxic compared to nicotine alone when added directly to human bronchial epithelial cells (Figure 1 B). This finding is in accordance with a previous study^32^. Aerosolized Smoothol in presence of nicotine showed significant growth inhibition of BEAS-2B cells (Figure 1 C). However, the aerosols from ‘rainbow candy’ and vanilla flavors showed moderate growth inhibition (Figure 1 D). In a previous investigation, we had identified the chemicals in all of the flavors (except Smoothol) selected for the current study: ethyl vanillin PG acetal (french vanilla, vanilla and ‘vanilla custard’);δ-tetradecalactone (vanilla and ‘vanilla custard’); Linalool (‘rainbow candy’); d-limonene (‘rainbow candy’); Piperonal (vanilla and ‘vanilla custard’); Ethyl maltol (french vanilla and ‘vanilla custard’); Neral (‘rainbow candy’); and γ-decalactone (‘rainbow candy’)^13^. The growth inhibition may be in part due to elevated levels of free radicals generated in the above flavors when tested as e-cigarette aerosols^13,33^. Other reports in BEAS-2B cells show similar growth inhibition by e-liquids, without flavors^34,35^ and with flavors^25,36,37^. Various flavored e-liquids also showed growth inhibition in U937 (monocytic cell line) cells^26,38^ directly exposed to e-liquids and/or aerosols at high doses^39^. Moreover, our study confirmed that BEAS-2B cells exposed to CSEs show a strong growth inhibition in a concentration-dependent manner consistent with other studies^25,38^ (Supplementary file Figure 5). We also validated that 3R4F is more toxic compared to Spectrum 102. Perhaps this difference could be attributed to tar, nicotine, and CO levels^40^.

We observed an overall reduced inflammatory response caused by flavors based on Z-scoring approach when we evaluated the combined impact on TNF-α, IL-6, IL-8 and MCP-1. Although, a systematic review compared several studies on the impact of flavors and/or nicotine on separate inflammatory response markers *in vitro*^41^, to our knowledge, this is the first study evaluating the effect of nicotine and/or flavors on combined inflammatory response using Z-scoring approach following aerosolized e-liquid exposure on THP-1 derived macrophages. Previously, investigators have observed increase in IL-6 and TNF-α^42^, and others have shown a decrease in TNF-α, IL-6 and MCP1, but an increase in IL-8^43^ following aerosolized e-liquid exposure in human alveolar macrophages as well as in THP-1 cells. Another study reported an increase in lung inflammation in a mouse model for allergic airway disease with flavored e-liquids without nicotine^44^. IL-8 has been reported to be induced by ortho-vanillin in BEAS-2B and fibroblasts^26,45^. Additionally, a study on epithelial cell cultures demonstrated that aerosolized nicotine exposure was sufficient to stimulate IL-6, IL-8 and MCP-1 release^46^. This indicates that multiple different inflammatory pathways may be activated by the various constituents of e-cigarette vapor, dependent on cell type, environment, and dose^47^.

Nicotine is also anti-inflammatory in our experimental conditions using THP-1 derived macrophages as reported earlier^43^. However, when considering CSEs, a low-dose inflammatory and high-dose anti-inflammatory response to TNF-α was observed in both 3R4F and Spectrum suggesting there may be additional components in the CSE contributing to the biphasic response. Overall, the inflammatory response is important for the primary defence of the lungs. It seems, various components of e-cigarettes including humectants, flavors and nicotine could contribute to overlapping inflammatory pathways.

Cigarettes do impact the basic property of macrophages to phagocytose and it would be informative to know the relative impact of e-cigarettes, and if flavors have an additional influence. In our study, we examined phagocytosis in macrophages and observed that Smoothol-containing aerosolized e-liquid, regardless of nicotine, reduced the phagocytic index of THP-1 derived macrophages. Other reports demonstrated similar reduction by e-cigarette aerosols without nicotine^44^. However, they used PMA as a stimulus instead of LPS and did not investigate flavors. Moreover, it was also demonstrated that e-cigarettes could cause macrophage efferocytosis dysfunction via reduced expression of apoptotic cell recognition receptors^27^. The reduced phagocytosis in macrophages could indicate that naïve e-cigarette users selecting flavored e-liquids might suffer from impaired bacterial clearance.

E-cigarettes and cigarette smoke induce oxidative stress^48^. Oxidative stress plays a key role in cigarette smoke-induced alveolar injury and REDD1 has been implicated in this process^11,49,50^. REDD1 makes lungs susceptible to tobacco smoke and destruction of alveoli leading to inflammation by activating NFκB and thereafter leading to increased expression of cytokines which in turn recruit neutrophils and macrophages^11^. In other words, REDD1 is necessary and sufficient in amplifying oxidative stress caused by tobacco smoke and is mediated by activation of NOX4 causing increased ROS^50^. On the other hand, REDD1 protects dividing cells from hypoxia or H_2_O_2_-induced apoptosis, while it sensitizes differentiated cells to stress^51,52^ and there may be another puzzling issue that REDD1 function appears sometimes beneficial, sometimes harmful in the progression and the physiopathology of metabolic diseases^53^. REDD1 is also an endogenous inhibitor of mTOR^54^. It is yet not clear if REDD1 is playing a role in causing potential harm in the lungs of e-cigarette users. In our study, the degree of increase in REDD1 for e-liquid was much lower compared to CSE. Additionally, we noticed that the cells treated with Smoothol flavored aerosolized e-liquid had higher expression of REDD1 compared to unflavored aerosolized e-liquid. Further investigations are required to evaluate several flavored e-liquids in order to propose REDD1 as a potential marker for flavor-induced toxicity in the lung. Moreover, flavors may be causing oxidative stress and could be linked to release of free radicals as reported in another study^13^ which emphasized the role of flavors in lipid peroxidation and 8-isoprostane formation. Moreover, cell free reactive oxygen species were reported to be produced using a variety of e-cigarette flavors and their individual components as well as mixed flavors^38^.

### Strengths and limitations

Our study goal was to evaluate cytotoxicity and inflammatory response in a variety of e-liquids with and without flavors. We showed a measurable impact of 1% aerosolized e-liquids on inflammatory milieu *in vitro* in three of the five flavors tested. REDD1 was identified as a potential biomarker for oxidative response in Smoothol-flavored e-liquid. The *in vitro* model set up for cytotoxicity and inflammatory response biomarkers could clearly assess readout for various e-liquids tested. The study has limitations. We only tested five of the prominent flavors on the market. It is important to keep in mind that often e-cigarette users will switch back and forth between cigarettes and e-cigarettes, and could also be dual users. We have not examined these scenarios but warn that dual use of e-cigarettes and cigarettes may be even more harmful to health^55^. In a future study, we intend to use a non-observed adverse effect level approach to expand on the biomarkers of harm in the normal bronchial epithelial cell population.

## CONCLUSIONS

Our *in vitro* bioassays can be utilized to effectively measure flavor and nicotine-induced effects of e-cigarettes on combined inflammatory response and cytotoxicity in human macrophages and human bronchial epithelial cells, respectively. Our results showed that 1% of aerosolized e-liquids containing french vanilla, ‘vanilla custard’ and ‘rainbow candy’ as well as Smoothol flavors, reduced the combined inflammatory response markers in THP-1 derived macrophages. Although this outcome might pose greater potential harm to infected individuals who are naïve e-cigarette users, e-liquids are generally less cytotoxic to the growth of bronchial epithelial cells when compared to cigarette smoke extracts on an equivalent puff basis. REDD1, may serve as a potential biomarker following further experimentation. While further research is needed to fully understand the effects of e-cigarette exposure in humans, we caution against the widely held opinion that e-cigarettes are safe.

## Data Availability

The data supporting this research can be found in the Supplementary file.
