# Supplementary material for: Evaluating electronic cigarette cytotoxicity and inflammatory responses in vitro
Source: Tob Induc Dis. 2022 May 10;20:45. doi: 10.18332/tid/147200 (PMC9081552; doi:10.18332/tid/147200)
Supplement: Supplementary file 1 [file TID-20-45-s1.pdf]

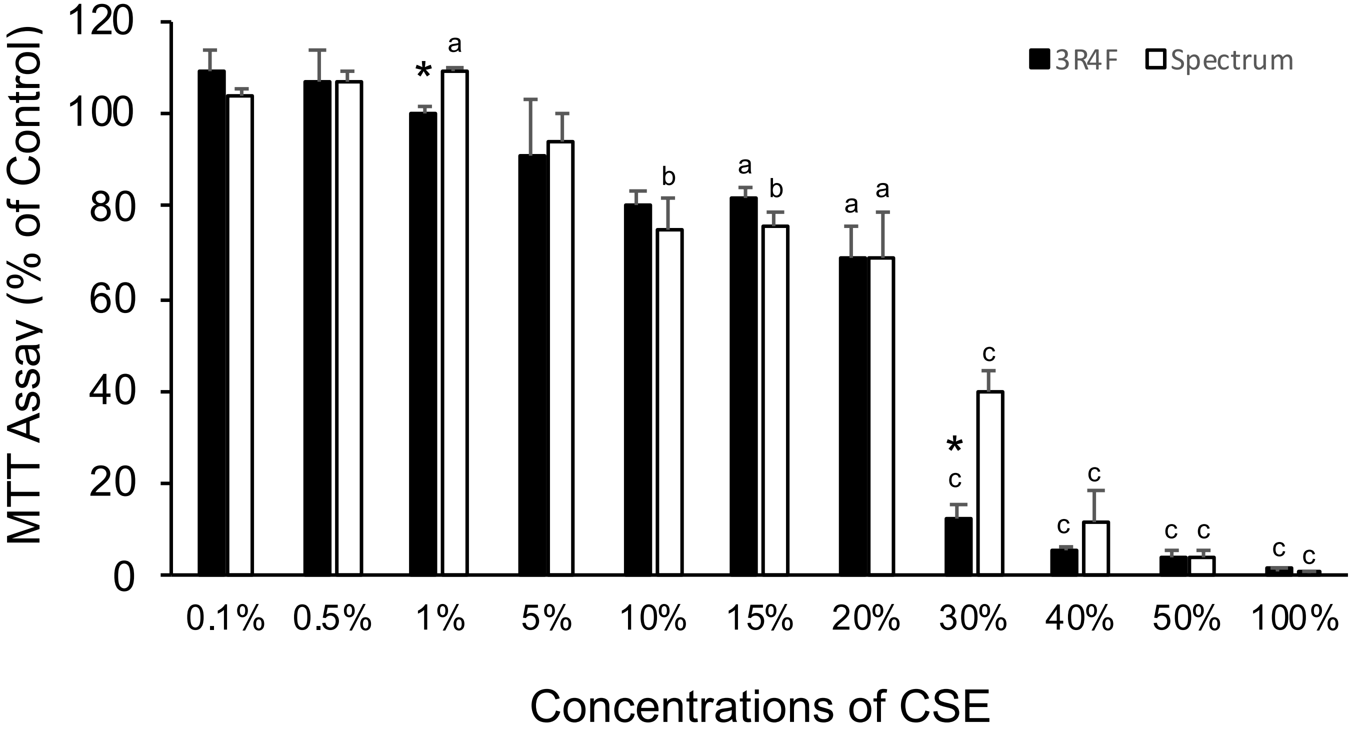

Supplementary Figure 1. Cytotoxicity of CSE measured by MTT assay. Various concentrations of CSE from 3R4F and Spectrum were incubated with BEAS-2B cells for 24 h and showed a reduced growth in a dose-dependent manner. 3R4F was found to be more cytotoxic than Spectrum at 30% CSE. ^{a} P<0.05, ^{b} P<0.01, ^{c} P<0.001 compared to control, * P<0.01 comparing 3R4F with Spectrum CSE.

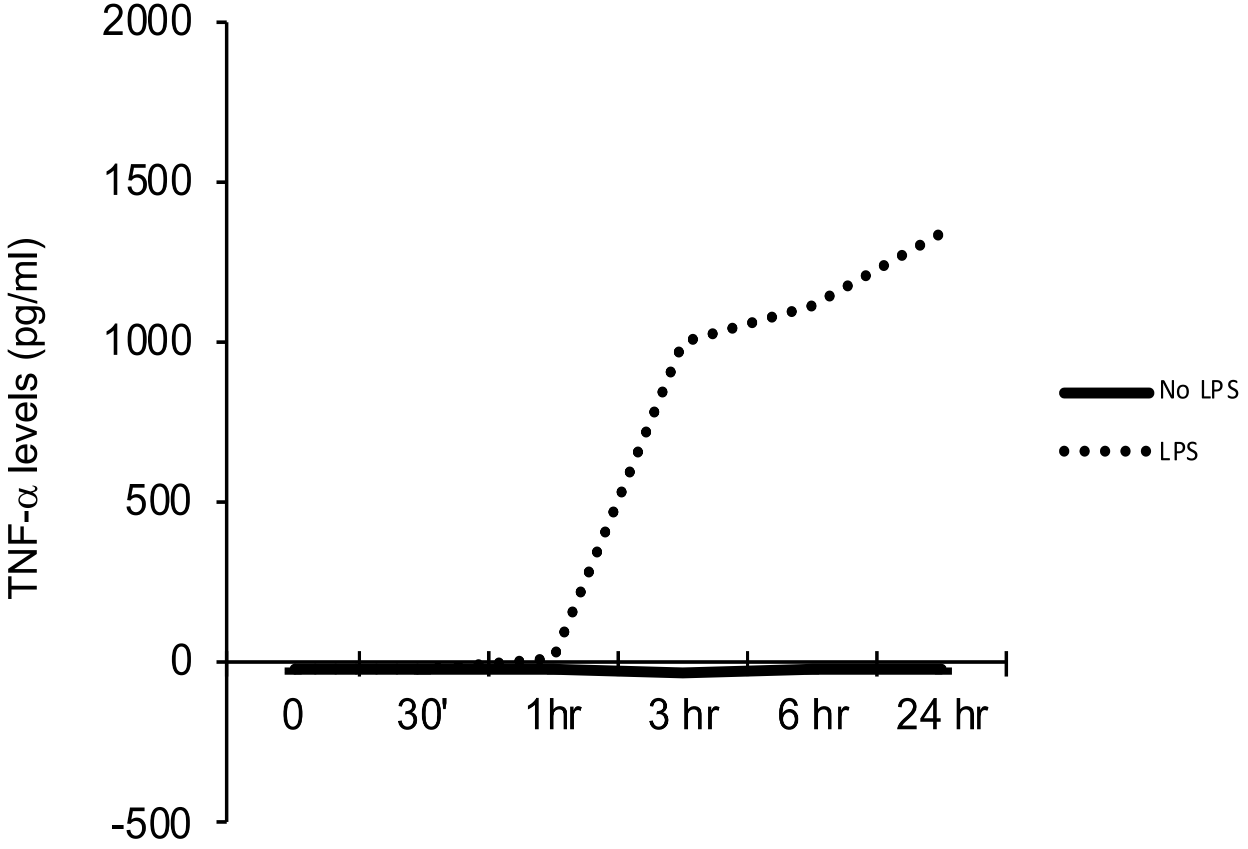

Supplementary Figure 2. Standardizing time for measuring TNF-α in THP-1 Macrophages in response to LPS (100 ng/ml).

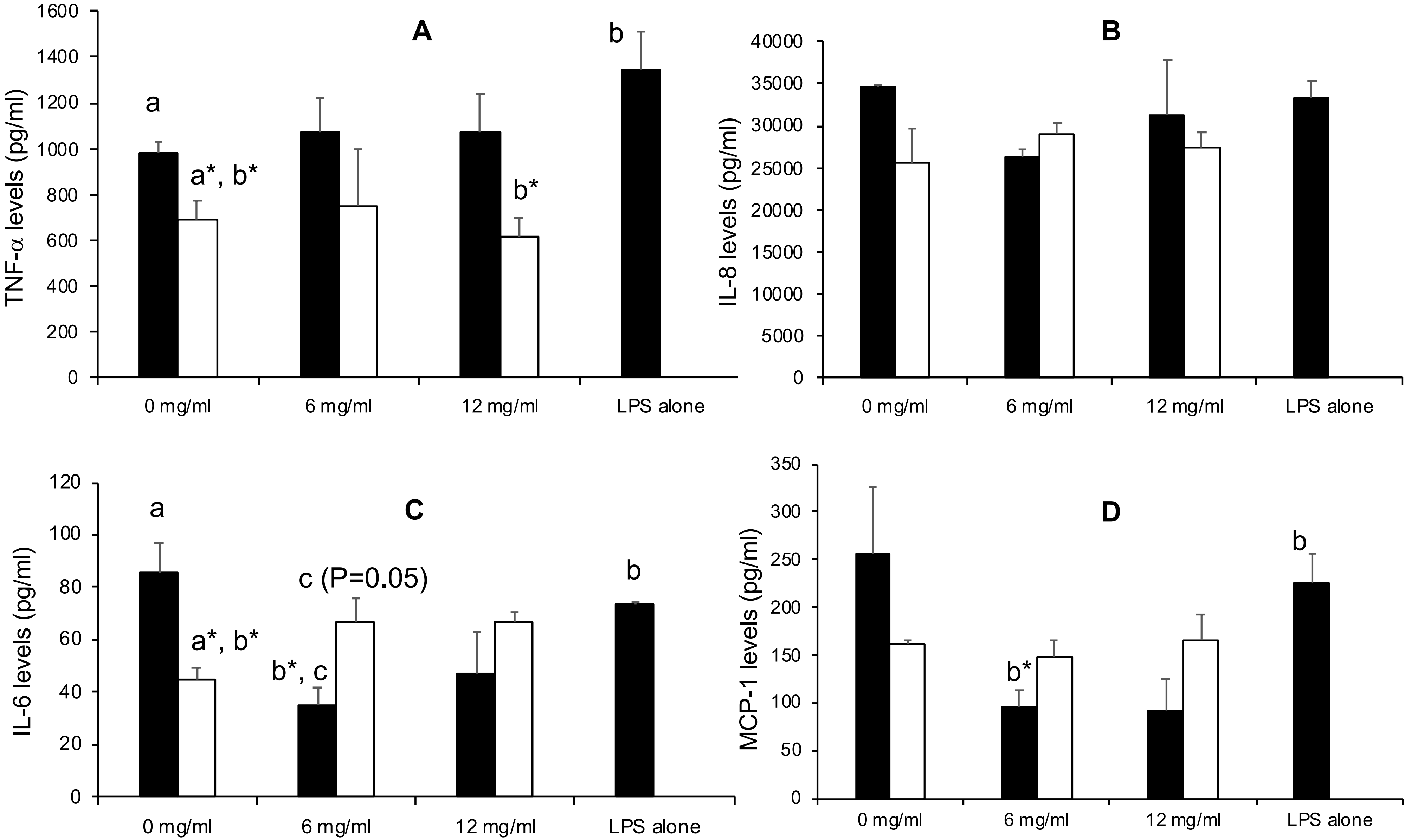

Supplementary Figure 3. Inflammatory response biomarkers in THP-1 derived macrophages following incubation with 1% aerosolized nicotine containing e-liquids. TNF-α, IL-8, IL-6 and MCP-1 levels in no flavor or Smoothol flavored nicotine containing e-Liquids differed. Mainly, IL-6 and MCP-1 levels were higher in Smoothol exposed macrophages while TNF-α were reduced by Smoothol in presence of nicotine. Black rectangle: Unflavored, white rectangle: Smoothol Flavor. Comparisons are indicated as vs a (PG:VG), b (LPS alone) or c (6 mg/ml nicotine with and without Smoothol), * P<0.05.

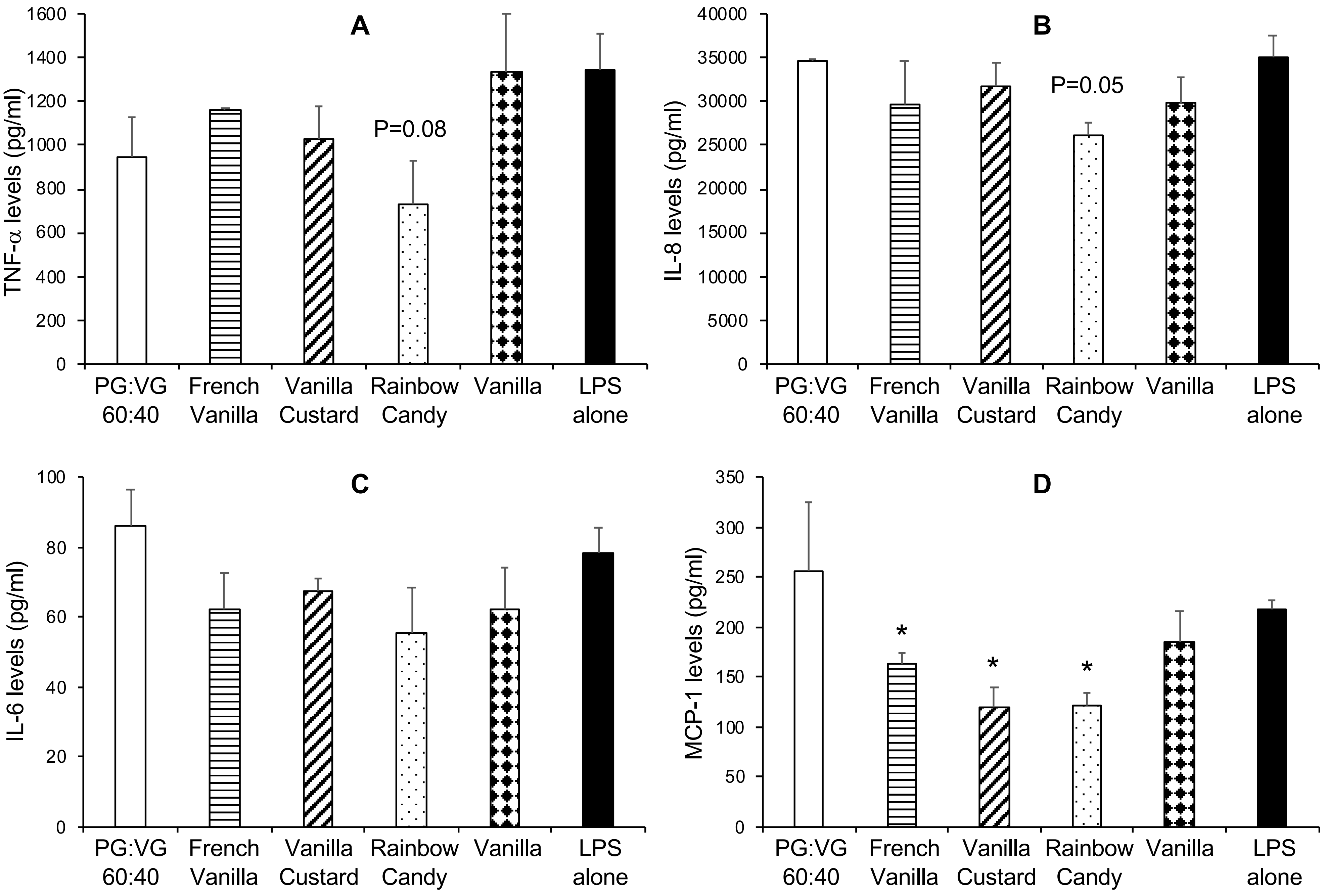

Supplementary Figure 4. Inflammatory marker response following treatments with 1% aerosolized flavored e-liquids in THP-1 derived macrophages. Rainbow Candy showed a non-significant drop in TNF-α and IL-8 levels but a significant decrease in MCP-1 when compared to LPS alone. French Vanilla and Vanilla Custard showed significant reduction in MCP-1 levels when compared to LPS alone, * $P<0.05$ compared to LPS alone.

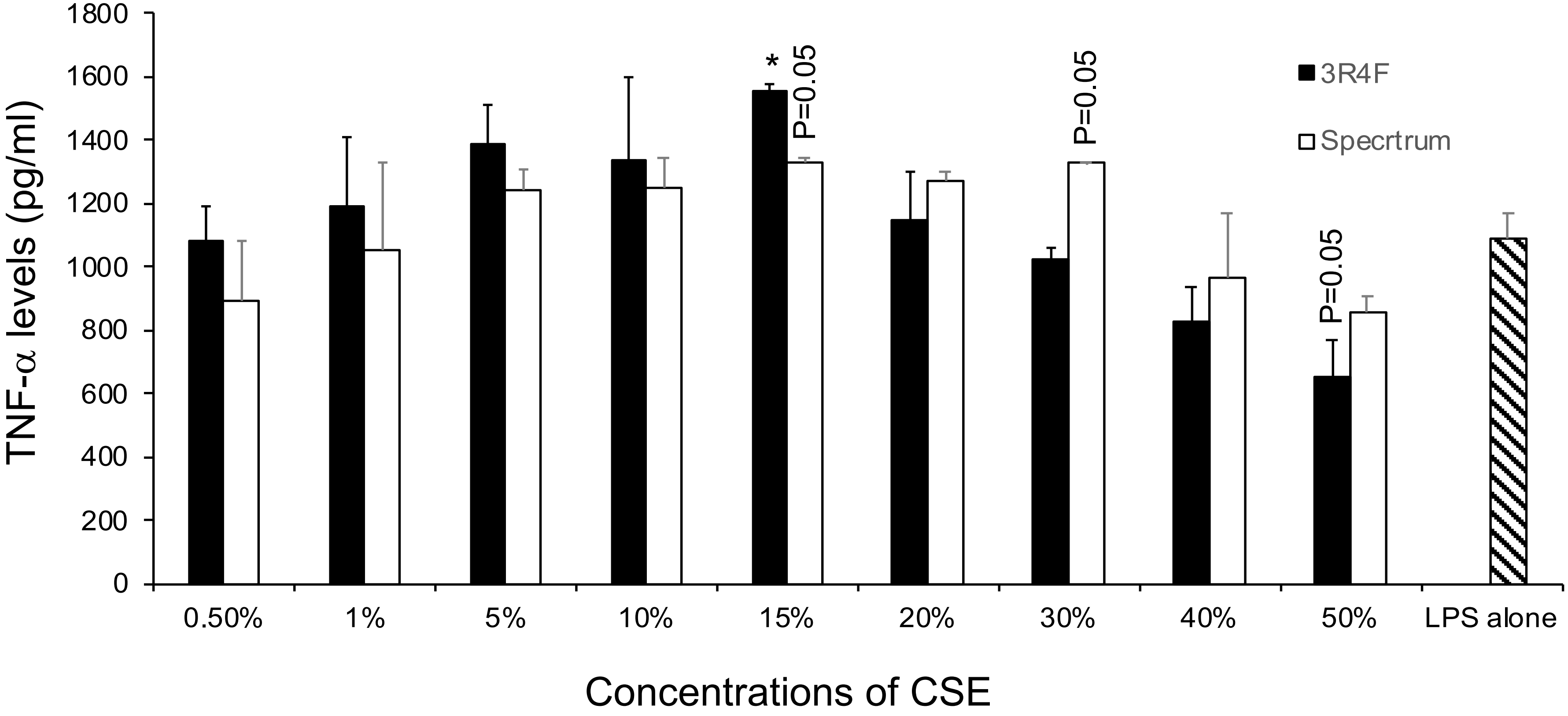

Supplementary Figure 5. Inflammatory response of CSE. 3R4F and Spectrum smoke extracts were inflammatory as observed by rising trend of TNF-α up to 15% and 30% CSE respectively and thereafter the effect seemed anti-inflammatory at increasing concentrations of 3R4F and Spectrum CSE in the presence of LPS. The statistical comparisons were done against LPS alone treatments, * $P<0.05$.

© 2022 Sinha I. et al.
