# Supplementary material for: Evaluating electronic cigarette cytotoxicity and inflammatory responses in vitro
Source: Tob Induc Dis. 2022 May 10;20:45. doi: 10.18332/tid/147200 (PMC9081552; doi:10.18332/tid/147200)
Supplement: Supplementary file 2 [file TID-20-45-s2.pdf]

Plain Language Summary

Please note that this summary only contains information from the full scientific article:

View Scientific Article

How effective are stop-smoking medicines for people with smoking-related conditions?

Bupropion
<byoo-PROH-pee-on>

Nicotine
<NIH-kuh-TEEN>

Varenicline
<vuh-REH-nih-klin>

Date of summary: November 2021

Study number: NCT01456936

Study start date: November 2011

Study end date: January 2015

The full title of this article: Medication-assisted quit rates in participants with smoking-related diseases in EAGLES: post hoc analyses of a double-blind, randomized, placebo-controlled clinical trial

Key takeaway

- In this study, Chantix/Champix (varenicline), bupropion, and nicotine replacement therapy (nicotine patch) worked well in helping people with smoking-related conditions to stop smoking.
- Side effects with these medicines were experienced at a similar level in smokers with and without smoking-related conditions.

The purpose of this plain language summary is to help you to understand the findings from recent research.

- Varenicline, bupropion, and nicotine replacement therapies are approved to help people stop smoking.
- The results of this study may differ from those of other studies. Health professionals should make treatment decisions based on all available evidence and not on the results of a single study.

More information can be found in the scientific article of this study, which you can access here: View Scientific Article

Additional information

More information can be found in the scientific article of this study, which you can access here:

View Scientific Article

For more information on this study, please visit:

https://clinicaltrials.gov/ct2/show/NCT01456936

For more information on clinical studies in general, please visit:

https://www.clinicaltrials.gov/ct2/about-studies/learn

Bupropion
<byoo-PROH-pee-on>

Nicotine
<NIH-kuh-TEEN>

Varenicline
<vuh-REH-nih-klin>

What did this study look at?

- Smoking can increase the risk of developing smoking-related conditions including:
  - Asthma, a lung condition caused by swelling of the breathing tubes;
  - Chronic obstructive pulmonary disease, a group of lung conditions that cause breathing difficulties;
  - Cardiovascular diseases, a group of conditions that affect the heart and blood vessels; and
  - Diabetes, a condition that causes the level of sugar in the blood to become too high.
- Helping smokers to stop smoking is important. It lowers the risk of death and disability caused by smoking-related conditions.
- When people smoke, they become addicted to a chemical called nicotine.
- Varenicline, bupropion, and nicotine replacement therapies are medicines that help people to stop smoking.
  - People take varenicline and bupropion as tablets. The medicines act on different types of receptors in the brain that play a part in addiction to nicotine.
  - They work in a similar way to nicotine and help reduce the cravings and withdrawal symptoms that people experience when they stop smoking.
    - While taking varenicline, smoking cigarettes can be less enjoyable.
  - Nicotine patches are a type of nicotine replacement therapy that people apply to the skin. They allow a small amount of nicotine to enter the body without the added chemicals found in cigarettes.
- In the EAGLES study, researchers looked at how safe and effective varenicline, bupropion, and nicotine patches were in smokers with and without mental health problems.
  - All 3 medicines helped people to stop smoking, but more people stopped smoking when they took varenicline.
- Few studies have looked at how well the medicines work for people with different smoking-related conditions.
- In this study, researchers looked at how effective varenicline, bupropion, and nicotine patches were in smokers with smoking-related conditions who took part in EAGLES. They looked at:
  - The number of people who stopped smoking while using the medicines
  - If people had anything about them (such as where they lived, or what medicine they took during the study) that could help to predict if they were likely to stop smoking
  - Any side effects that people experienced during the study.
    - A side effect is something (expected or unexpected) that a person feels was caused by a medicine or treatment they take.

Additional information

More information can be found in the scientific article of this study, which you can access here:

View Scientific Article

For more information on this study, please visit:

https://clinicaltrials.gov/ct2/show/NCT01456936

For more information on clinical studies in general, please visit:

https://www.clinicaltrials.gov/ct2/about-studies/learn

Bupropion
<byoo-PROH-pee-on>

Nicotine
<NIH-kuh-TEEN>

Varenicline
<vuh-REH-nih-klin>

Where is this study in the drug development timeline?

- Varenicline, bupropion, and nicotine patches are approved treatments to help people to stop smoking.

Clinical trial phases

Phase 1
Healthy volunteers take a medicine so researchers can look at how safe it is and how it works in the body

Phase 2
A small number of patients with a specific condition take a medicine to see how well it works, what dose should be taken, and how safe it is

Phase 3
A large group of patients with a specific condition take a medicine to see how well it works and how safe it is

Approval
All of the results are reviewed by agencies that approve medicines and, if approved, the medicine is made available for doctors to prescribe to patients, or for patients to buy

Phase 4
After a medicine is approved, researchers do studies in different groups of people not included in earlier trials, and over long periods of time, to monitor how effective and safe it is

EAGLES is a Phase 4 clinical trial

Who took part in this study?

- Adults aged 18–75 years old from 16 countries took part in EAGLES.
- They could take part if they smoked at least 10 cigarettes every day and they wanted to stop smoking.

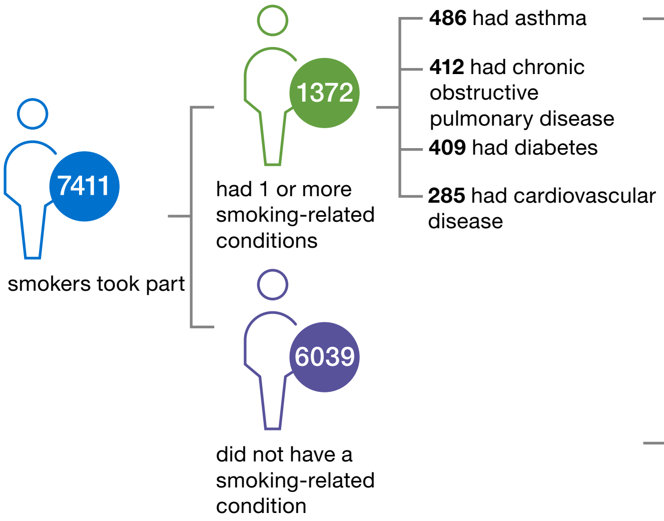

- Researchers randomly assigned smokers to take **varenicline**, **bupropion**, **nicotine patch**, or a **placebo**.
  - A placebo looks like the study drug but has no active ingredients.
- All smokers received brief counselling while receiving treatment to help them to stop smoking.

12 weeks → Smokers took their medicine or placebo for 12 weeks

12 weeks → Researchers recorded peoples' smoking habits for 12 weeks following treatment

Additional information

More information can be found in the scientific article of this study, which you can access here:

View Scientific Article

For more information on this study, please visit:

https://clinicaltrials.gov/ct2/show/NCT01456936

For more information on clinical studies in general, please visit:

https://www.clinicaltrials.gov/ct2/about-studies/learn

Bupropion
<byoo-PROH-pee-on>

Nicotine
<NIH-kuh-TEEN>

Varenicline
<vuh-REH-nih-klin>

- On average, people with a smoking-related condition were 52 years of age.
- On average, people without a smoking-related condition were 45 years of age.
- People with smoking-related conditions had smoked for an average of 34 years.
- Otherwise healthy smokers (smokers without smoking-related conditions) had smoked for an average of 27 years.

What were the results of the study?

- Compared with otherwise healthy smokers, people with smoking-related conditions:
  - Were older
  - Had smoked for longer
  - Had tried to stop smoking more often.
- They were also more likely to:
  - Have mental health problems
  - Live in the United States
  - Have used stop-smoking medicines before.

How many people stopped smoking while taking the medicines?

- Researchers recorded the percentage of people who stopped smoking completely for a certain period of time.

Researchers looked at:

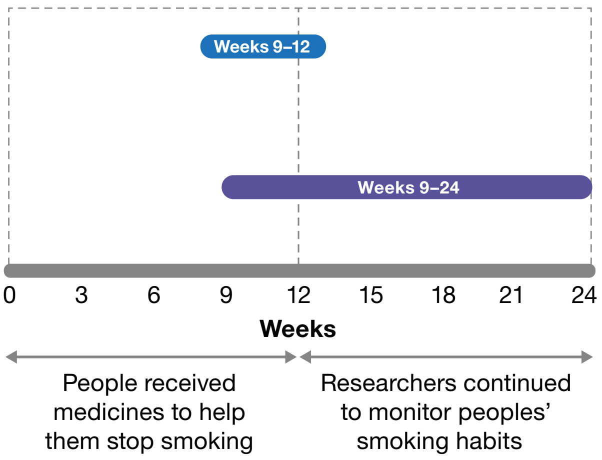

4 weeks: The percentage of people who stopped smoking for the last 4 weeks of treatment **(weeks 9–12)**

16 weeks: The percentage of people who stopped smoking for the last 4 weeks of treatment plus 12 weeks after treatment stopped **(weeks 9–24)**

- The percentage of people who stopped smoking for weeks 9–12 and weeks 9–24 was lower for those with smoking-related conditions compared with otherwise healthy smokers.
  - When researchers looked at each smoking-related condition separately, this difference didn't apply to:
    - Smokers with diabetes at weeks 9–12 or weeks 9–24
    - Smokers with cardiovascular disease at weeks 9–12.

Additional information

More information can be found in the scientific article of this study, which you can access here:

View Scientific Article

For more information on this study, please visit:

https://clinicaltrials.gov/ct2/show/NCT01456936

For more information on clinical studies in general, please visit:

https://www.clinicaltrials.gov/ct2/about-studies/learn

Bupropion
<byoo-PROH-pee-on>

Nicotine
<NIH-kuh-TEEN>

Varenicline
<vuh-REH-nih-klin>

Percentage of people who stopped smoking for:

	Weeks 9–12	Weeks 9–24
People without a smoking-related condition	24%	17%
People with a smoking-related condition	21%	13%

Researchers also looked at the percentage of people with each smoking-related condition who stopped smoking

	Weeks 9–12	Weeks 9–24
Asthma	17%	11%
Chronic obstructive pulmonary disease	18%	10%
Diabetes	24%	17%
Cardiovascular disease	25%	12%

- More people stopped smoking if they were taking varenicline or bupropion or using nicotine patches than if they were taking placebo. This included people with and without a smoking-related condition.
- More people (with or without a smoking-related condition) stopped smoking if they were taking varenicline than if they were taking bupropion or using nicotine patches.
  - Among smokers with each of the different smoking-related conditions, more people stopped smoking if they were taking varenicline than if they were taking bupropion or using nicotine patches.
    - The percentage of people who stopped smoking for weeks 9–24 was similar for smokers with asthma if they took varenicline or bupropion.
- People took their medicine for a similar length of time:
  - Whether or not they had a smoking-related condition; and
  - Whichever medicine they were taking.

What factors may help to predict whether people will be able to stop smoking?

- Researchers used mathematical models to see if people had anything about them at the start of the study that may help predict if they were more likely to stop smoking. These included:
  - If they had a smoking-related disease
  - If they had mental health problems
  - Which medicine they took in the study
  - Where they lived.
- Taking a medicine to help them stop smoking was the most useful factor for predicting which people would stop smoking.

Additional information

More information can be found in the scientific article of this study, which you can access here:

View Scientific Article

For more information on this study, please visit:

https://clinicaltrials.gov/ct2/show/NCT01456936

For more information on clinical studies in general, please visit:

https://www.clinicaltrials.gov/ct2/about-studies/learn

Bupropion
<byoo-PROH-pee-on>

Nicotine
<NIH-kuh-TEEN>

Varenicline
<vuh-REH-nih-klin>

Compared with people taking placebo:

People taking **varenicline** were	People taking **bupropion** were	People using **nicotine patch** were
4×	2×	2×

more likely to have quit during weeks 9–12

People taking **varenicline** were	People taking **bupropion** were	People using **nicotine patch** were
3×	2×	2×

more likely to have quit during weeks 9–24

- People were more likely to stop smoking for weeks 9–12 and 9–24 if they lived outside the United States, and were of older age.
- Having tried to stop smoking before helped to predict that people would stop smoking for weeks 9–12, but not for longer periods (weeks 9–24).
- People with a smoking-related condition or with mental health problems were less likely to stop smoking.

What were the side effects of the treatments?

Side effects experienced by at least 5 in 100 people with or without a smoking-related condition were:

- Urge to vomit
- Difficulty sleeping or strange dreams
- Anxiety
- Tiredness
- Dry mouth
- Headache
- Cold symptoms
- Infections of the sinuses or throat
- Dizziness

Additional information

More information can be found in the scientific article of this study, which you can access here:

View Scientific Article

For more information on this study, please visit:

https://clinicaltrials.gov/ct2/show/NCT01456936

For more information on clinical studies in general, please visit:

https://www.clinicaltrials.gov/ct2/about-studies/learn

Bupropion
<byoo-PROH-pee-on>

Nicotine
<NIH-kuh-TEEN>

Varenicline
<vuh-REH-nih-klin>

- In this study, 4 in every 100 people with and without a smoking-related condition reported a problem related to their mental health.
- Researchers weighed people at the start of the study, at week 12, and at week 24.
  - At week 12 and week 24, the average amount of weight that people gained was less than 2 kg, whether or not they had a smoking-related condition and whichever medicine they took to help them stop smoking.

More results from this study can be found here: **View Scientific Article**

What were the main conclusions reported by the researchers?

- People who had already developed certain smoking-related conditions were less likely to stop smoking than those without those conditions.
- Varenicline, bupropion, and nicotine patches were effective and safe in helping people with smoking-related conditions to stop smoking.
  - More people stopped smoking when they took varenicline than those who took bupropion or used nicotine patches.
- Taking one of the medicines was the best predictor of whether people would stop smoking during weeks 9–12 and weeks 9–24.
- Side effects while taking medicines to help stop smoking were similar in people with and without smoking-related conditions.
- The results from this study show that people with smoking-related conditions may benefit from taking medicines to help them stop smoking. This may be helpful for smokers and their doctors when making treatment decisions.

Who sponsored this study?

Pfizer Inc

235 East 42nd Street New York, NY 10017

Phone (United States): +1 212-733-2323

GlaxoSmithKline

5 Crescent Drive, Philadelphia, PA 19112

Phone (United States): +1 888-825-5249

The sponsors would like to thank all of the people who took part in this study.

Additional information

More information can be found in the scientific article of this study, which you can access here:

View Scientific Article

For more information on this study, please visit:

https://clinicaltrials.gov/ct2/show/NCT01456936

For more information on clinical studies in general, please visit:

https://www.clinicaltrials.gov/ct2/about-studies/learn

Acknowledgements
Writing support for this summary was provided by Katy Beck, PhD, at Engage Scientific Solutions, and was funded by Pfizer.
